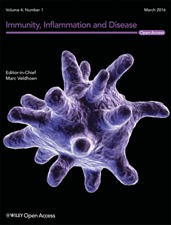# Issue Information

**DOI:** 10.1002/iid3.81

**Published:** 2016-02-26

## Abstract